# Tradeoff between Stem Hydraulic Efficiency and Mechanical Strength Affects Leaf–Stem Allometry in 28 *Ficus* Tree Species

**DOI:** 10.3389/fpls.2017.01619

**Published:** 2017-09-20

**Authors:** Ze-Xin Fan, Frank Sterck, Shi-Bao Zhang, Pei-Li Fu, Guang-You Hao

**Affiliations:** ^1^Key Laboratory of Tropical Forest Ecology, Xishuangbanna Tropical Botanical Garden, Chinese Academy of Sciences Mengla, China; ^2^Forest Ecology and Forest Management Group, Department of Forestry, Wageningen University Wageningen, Netherlands; ^3^Key Laboratory of Economic Plants and Biotechnology, Kunming Institute of Botany, Chinese Academy of Sciences Kunming, China; ^4^Key Laboratory of Forest Ecology and Management, Institute of Applied Ecology, Chinese Academy of Sciences Shenyang, China

**Keywords:** biomass allocation, *Ficus*, theoretical hydraulic conductivity, leaf–stem relationship, mechanical strength, trade-off, wood density

## Abstract

Leaf–stem allometry is an important spectrum that linked to biomass allocation and life history strategy in plants, although the determinants and evolutionary significance of leaf–stem allometry remain poorly understood. Leaf and stem architectures – including stem area/mass, petiole area/mass, lamina area/mass, leaf number, specific leaf area (LA), and mass-based leafing intensity (LI) – were measured on the current-year branches for 28 *Ficus* species growing in a common garden in SW China. The leaf anatomical traits, stem wood density (WD), and stem anatomical and mechanical properties of these species were also measured. We analyzed leaf–stem allometric relationships and their associations with stem hydraulic ad mechanical properties using species-level data and phylogenetically independent contrasts. We found isometric relationship between leaf lamina area/mass and stem area/mass, suggesting that the biomass allocation to leaf was independent to stem size. However, allometric relationship between LA/mass and petiole mass was found, indicating large leaves invest a higher fractional of biomass in petiole than small ones. LI, i.e., leaf numbers per unit of stem mass, was negatively related with leaf and stem size. Species with larger terminal branches tend to have larger vessels and theoretical hydraulic conductivity, but lower WD and mechanical strength. The size of leaf lamina, petiole, and stem was correlated positively with stem theoretical hydraulic conductivity, but negatively with stem WD and mechanical strength. Our results suggest that leaf–stem allometry in *Ficus* species was shaped by the trade-off between stem hydraulic efficiency and mechanical stability, supporting a functional interpretation of the relationship between leaf and stem dimensions.

## Introduction

Leaf surface area spans six orders of magnitude across terrestrial plants species (Milla and Reich, 2007; Díaz et al., 2016), and the functional and evolutionary significance of this diverse feature is a subject of strong interest (Ackerly and Reich, 1999). Leaves intercept light and acquire carbon, while stems transport water with nutrients and mechanically support the leaves, thus leaf and stem traits are expected to be highly coordinated biomechanically and physiologically (Westoby et al., 2002). At twig level, traits such as twig size, wood density (WD), or internode length have been reported to account for cross-species variation in leaf size (Westoby and Wright, 2003; Poorter and Rozendaal, 2008). Corner (1949) identified two basic properties of plant architecture: larger leaves are borne on thicker stems, and plants with thicker stems branch more sparsely, and vice versa.

This “Corner’s rules” has been tested empirically within and across species and environments (White, 1983a,b; Ackerly and Donoghue, 1998; Brouat et al., 1998; Olson et al., 2009). Positive allometric relationships between twig and leaf size are often found across species in different vegetation types (Westoby and Wright, 2003; Preston and Ackerly, 2003; Sun et al., 2006; Yang et al., 2010) and taxonomic groups (White, 1983a,b; Ackerly and Donoghue, 1998; Brouat et al., 1998; Normand et al., 2008), which implies that larger leaves need disproportionally more in supporting and transporting structures than smaller ones (Niinemets et al., 2006). However, isometric relationships have also been observed between twig and leaf size (e.g., Brouat et al., 1998; Li et al., 2008; Zhang et al., 2016). Therefore, the exact nature of this relationship and why it should occur are far from conclusive and the mechanism underlying Corner’s rules needs further investigations (Olson et al., 2009).

The leaf–stem size spectrums are often expected partly as a result of the mechanical and conductive demands of leaves (Niklas, 1994; Enquist, 2002), i.e., larger leaves would require a larger cross-sectional area for water supply and greater mechanical support (Shinozaki et al., 1964). Normand et al. (2008) suggested the leaf–stem allometry in mango cultivars is linked to the trade-off between hydraulic efficiency and mechanical strength of wood, supporting a functional interpretation leaf–stem size spectrum. However, it is not so obvious why stem cross-sectional area should scale positively with mean leaf size (Smith et al., 2017).

Plant xylem plays three main functions, i.e., mechanical support, water conduction, and water and photosynthate storage (Bittencourt et al., 2016). Vessel-bearing angiosperms allocated limited xylem space and resources for building different structures that acting different function, i.e., vessels transport water, fibers provide mechanical support, and parenchyma cells function for storage (Baas et al., 2004). The conflicting structural requirements in xylem design would lead a “trade-off triangle” among mechanical strength, conductive efficiency, and resistance to embolism (Sperry et al., 2008; Brodersen, 2016). An increase of leaf surface area requires the increases of stem conductive area or efficiency for water supply, and meanwhile increases of strength for mechanical loads. However, these conflicting demands cannot be accomplished unless plants adjust their xylem structure designs. It is still unclear that how xylem hydraulic and mechanical properties varied with stem/leaf dimensions, and if these changes could shape the stem–leaf size relationships.

In the study, we measured leaf, stem, and petiole dimensions (area, mass, and anatomical and mechanical properties) of terminal branches of 28 *Ficus* species growing in a common garden in tropical China. We analyzed the allometric relationships among these traits, both across species and across phylogenetically independent contrasts (PICs). We asked the questions: (1) are the scaling relationships between leaf size and stem/petiole size isometry or allometry? (2) Can the adjustments of plant xylem structures along with leaf/stem dimensions explain scaling relationships between leaves and stem size. We tested the specific hypothesis that there is a trade-off between hydraulic and mechanical function of stems, and this trade-off has compensated effect on the twig–leaf size allometric relationship.

## Materials and Methods

### Study Site and Species

This study was conducted at Xishuangbanna Tropical Botanical Garden (XTBG, 21°41′ N, 101°25′ E, and altitude 570 m), Yunnan, Southwest China. The region is dominated by warm, wet air masses from the Indian Ocean in summer and continental air masses from temperate regions in winter. The mean annual temperature is 21.7°C, with the monthly mean temperature being 15.9°C during the coldest month (December) and 25.7°C during the warmest month (June). The annual rainfall is 1560 mm, with more than 80% occurring during the wet season from May to October.

We selected 28 tree species all belong to *Ficus* genus that commonly found in the rainforests of Xishuangbanna (Supplementary Table S1). All plants were grown in a common garden of XTBG with relatively open habitats and homogeneous soil, and have reachable from the ground or by using a ladder. We sampled three-to-five branches randomly from three individuals per species. All the branches were located at the outer surface of the plant’s crown. A total 250 branches were sampled from the 28 *Ficus* species. Samples were taken from May to September of 2010 and 2011, when seasonal shoot growth and leaf expansion have been completed.

### Twig Architecture

For each sampled branch, a current-year terminal (un-branched) shoot (here defined as “twig”) with no herbivore damage was selected for trait measurements. For each twig sample, leaf lamina, petiole, and stem were separated. Leaf numbers were counted and leaf area (LA) was measured with an area meter (Li-Cor 3000A; Lincoln, NE, United States). Stem and petiole diameters were measured with a vernier caliper. Twig stem, all leaf laminas, and petioles from a twig were oven dried at 70°C to constant weight, and their dry masses were weighted. Specific leaf area (SLA) was expressed as LA per unit of dry mass (m^2^ kg^-1^). The individual lamina area (mass) was calculated as the total area (mass) divided by leaf number. Individual petiole mass was calculated as the total petiole mass divided by leaf number. Leafing intensity (LI), a common developmental correlate of leaf size, was defined as leaf numbers per unit of stem mass (no. g^-1^).

#### Stem Anatomical and Biomechanical Properties

After removing the bark and pith with a razor blade, the fresh wood (FW) are weighted and wood fresh volume was measured the water displacement method. Wood saturated weight (SW) was determined after submerging under water for 48 h for rehydration. Wood samples were then over-dried for at least 48 h at 70°C to determine the dry weight (DW). Stem saturated water content (SWC) was calculated as: SWC = (FW - DW)/(SW – DW) × 100%. WD (g cm^-3^) was determined by dividing the dry mass by the volume of the samples.

We made transvers wood cross-sections with a microtome, stained the sections with safranin. We photographed sections with a digital camera mounted on microscope (Leica DM2500, Germany). We used ImageJ software^1^ to measure vessel major and minor diameter, as well as vessel density (VD). Since vessel is mostly elliptical, vessel diameter was calculated as *D* = [32(*ab*)^3^/(*a*^2^+*b*^2^)]^1/4^, where *a* and *b* are the major and minor axis dimensions, respectively (Lewis, 1992). Hydraulic weighted vessel diameter (*D*_h_) is calculated as *D*_h_ = [1/*n*Σ*D*^4^]^1/4^. VD (no. mm^-2^) was calculated as number of vessels per unit of xylem area (mm^-2^). We calculated vessel fraction (VF) as the product of vessel area and VD. The theoretical specific hydraulic conductivity (*K*_theo_, kg m^-1^ MPa^-1^ s^-1^, a measure of xylem porosity) was estimated according to the Hagen–Poiseuille equation: *K*_theo_ = μρ/(128μ*A*_s_)[Σ*D*^4^], where ρ is the density of water (998.2 kg m^-3^ at 20°C), μ is the viscosity of water (1.002 × 10^-9^ MPa s^-1^ at 20°C), and *A*_s_ is the cross-sectional area of sapwood (Tyree and Zimmermann, 2002). The calculated *K*_theo_ is substantially higher than actual conductivity as it ignores resistance of water flowing through the vessel walls. However, calculated *K*_theo_ appears to be a good proxy for conductivity because vessel walls contribute a relatively constant 56% to total resistance in conduits (Sperry et al., 2006).

The modulus of elasticity (MOE) of stem (Young’s modulus, MPa) was measured by three-point bending method with an INSTRON mechanical testing machine with a 5 kN load cell (INSTRON Corporation, Canton, MA, United States). Stem segments had length-to-diameter ratios of 25:1 to avoid share. Stem segments were placed on a steel frame apparatus at two supporting points at a precise span distance. Consecutive weights were added to a pannier suspended from the exact center of the stem segment. Flexural stiffness (EI) and structural Young’s modulus (MOE) were calculated from linear relationships resulting from observed deflections of stem segments after adding a sequence of weights.

### Leaf Anatomy

For each sampled branch, three to five full-developed leaves were sampled from twigs closed to those used for measuring twigs architecture. Hand transverse sections were made for leaves and then mounted on glass slides, and then these sections were examined and photographed at 10× magnifications with a light microscope (Leica DM2500, Germany). Total leaf thickness (LT), thickness of the upper epidermis (UET, mm), lower epidermis (LET, mm), palisade tissue (PT, mm), and spongy tissue (SP, mm) were measured with ImageJ software. Stomatal size (SS) was represented as the guard cell length and stomatal density (SD) was calculated as the number per unit LA.

### Statistical Analysis and Comparative Methods

Results from hierarchical ANOVA revealed that trait variations among species consistently the largest, i.e., explained 78.6–98.8%. Trait variations among individuals from the same species, and among twigs from the same individual, were mostly less than 20% (Supplementary Table S2). For interspecific comparisons, trait values were averaged arithmetically per individual and then per species. The species mean trait values were log_10_-transformed to fit the normal distribution (Kerkhoff and Enquist, 2009).

The relationships among paired traits were described by a mathematical equation of the type *y* = γ *x*^β^, where *y* and *x* are variables or parameters.[AQ]edit They can be linearized through a logarithm transformation of both variables: *Y* = α + β*X*, where *Y* = log(*y*), α = log(γ), and *X* = log(*x*). The linear relationship is described by its slope β or scaling coefficient and its *y*-intercept α or allometric constant. The value of the slope determines the relationship is isometric (β = 1) or allometric (β ≠ 1). Standardized major axis (SMA) regression was used to estimate the parameters of the allometric equation, by using the R package “SMATR” (Warton et al., 2006).

We assessed relationships between traits using Pearson correlation for 24 traits of all the 28 *Ficus* species. We carried out a multiple factor analysis (MFA) for the measured stem and leaf traits, with the method of Escofier and Pages (1994). We performed the MFA for 13 leaf and 11 stem/petiole trait means of the 28 *Ficus* species, by using the FactoMineR package (Lê et al., 2008) in R v.3.23 (R Development Core Team, 2004).

To test if correlations resulted from phylogeny relatedness, we also calculated pairwise correlations using PICs (Felsenstein, 1985; Garland et al., 1992). PICs for the 24 functional traits were calculated based on a phylogeny tree of the 28 *Ficus* species, using the “ape” package (Felsenstein, 1985; Garland et al., 1992). The phylogenetic relationships among the 28 *Ficus* species were inferred from nuclear ITS and G3pdh sequences deposited in GenBank (Supplementary Table S1; Xu et al., 2011). Sequences alignment was performed using Clustal W default settings followed by a manual adjustment in the MEGA 5.2 software (Tamura et al., 2007). Phylogenetic tree was estimated using Bayesian methods with the MrBayes v. 3.2 (Supplementary Figure S1).

## Results

**FIGURE 1:**
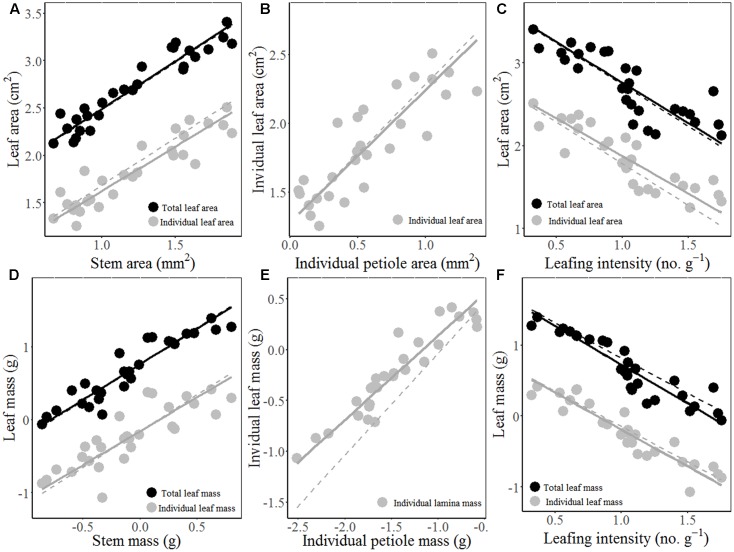
The scaling relationships between stem area and LA/mass **(A,D)**; individual petiole area/mass and lamina area/mass **(B,E)**, and mass-based LI and LA/mass **(C,F)** for 28 *Ficus* tree species. The solid lines are the standardized major axis (SMA) regression curves. The dashed lines are the slopes equal to 1.0 or –1.0.

The total and individual LA (mass) scaled positively with stem area (mass), with best-fit common regression slopes were not significantly different with slope 1.0 (**Figures 1A,D** and **Table 2**). Petiole area (mass) was highly related to lamina area (mass), with the scaling slopes between petiole and leaf mass were significantly smaller than 1.0, indicating an allometric scaling relationship between petiole and leaf lamina (**Figures 1B,E** and **Table 2**). Leaf size showed a negative, isometric relationship with mass-based LI, with the best-fit common regression slopes ranged from -0.9 to -1.1 (**Figure 1D**). A significant positive allometric relationship was found between lamina mass and lamina area, with common slopes (1.1–1.6) significantly departed from the value of 1.0 (**Table 2** and Supplementary Figure S2).

**FIGURE 2:**
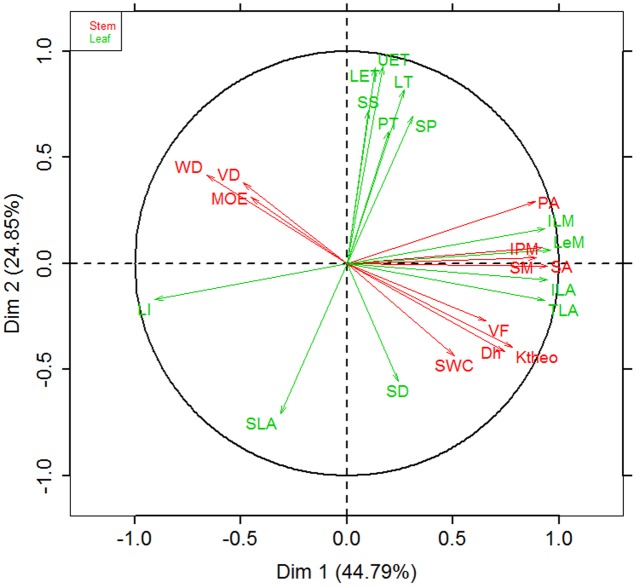
Biplot of trait relationships based on multiple factor analysis (MFA) on cross-species means of 11 stem/petiole (red) and 13 leaf traits (green) of 28 *Ficus* species. Trait abbreviations and unit of measure are shown in **Table 1**. Values in parentheses in the axis labels are percentages of variance explained.

The results of MFA revealed three trade-offs (**Figure 2**): i.e., (1) trade-off between stem hydraulic efficiency and mechanical strength; (2) trade-off between SLA and the thickness of all lamina tissues, (3) trade-off between leaf/stem size and LI. The first axis of MFA accounted for 44.79% of the total variance among the 24 traits, which loaded mainly the leaf architecture traits, i.e., stem area/mass, LA/mass, petiole area/mass, and mass-based LI (**Figure 2**). In other word, species with larger leaves have thicker stem and petiole, but lower mass-based LI. Meanwhile, the first axis also loaded positively the traits associated with stem hydraulic efficiency (i.e., *D*_h_, VF, *K*_theo_, and SWC), but loaded negatively the traits linked to stem mechanical strength (WD and MOE). The second axis accounted for 24.85% of the variance, and mainly loaded SLA and traits related to lamina tissue thickness (LT, UET, PT, SP, and LET) and SS and SD (**Figure 2** and Supplementary Figure S3). The results of the MFA based on PICs were generally consistent with that of cross-species means (Supplementary Figure S4 and Table S3).

**FIGURE 3:**
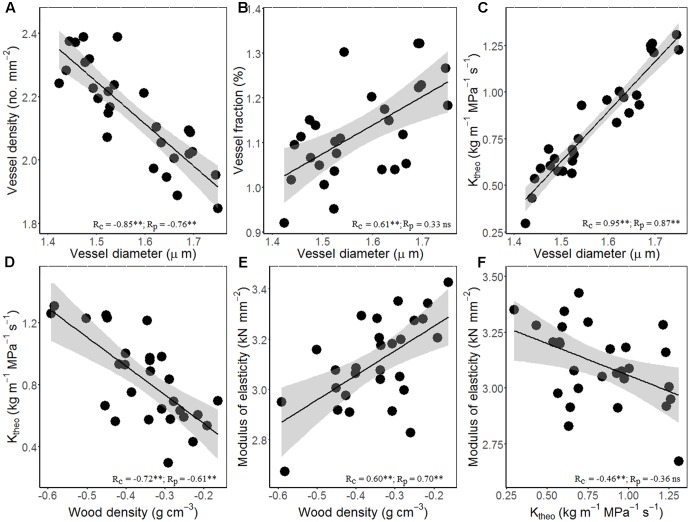
Relationships among stem hydraulic architectural traits, wood density (WD), and mechanical properties for 28 *Ficus* species. **(A)** Vessel diameter vs. density, **(B)** vessel diameter vs. fraction, **(C)** vessel diameter vs. theoretical hydraulic conductivity (*K*_theo_), **(D)** WD vs. *K*_theo_, **(E)** WD vs. modulus of elasticity (MOE), and **(F)** *K*_theo_ vs. MOE. Note the log_10_ scales in panels. Shading areas represent 95% confidence intervals of linear regression. Pearson’s cross-species (*R*_c_) and phylogenetically independent contrast (PIC) (*R*_p_) correlation coefficients are shown. *ns*, *p* > 0.05; ^∗^*p* < 0.05; ^∗∗^*p* < 0.01.

**Figure 3** showed the trade-offs between stem hydraulic efficiency and mechanical strength. Species with larger xylem vessel size (*D*_h_) had lower VD, but higher VF and thus high theoretical hydraulic conductivity (*K*_theo_) (**Figures 3A–C**). On the other hand, theoretical hydraulic conductivity (*K*_theo_) showed negative correlations with WD and MOE (**Figures 3D–F**). Thus, higher xylem hydraulic efficiency can only be achieved by the cost of reduced WD and mechanical strength. As compared with cross-species correlations, the correlations based on PICs were generally weaker, and in some case became non-significant (i.e., vessel diameter vs. fraction and *K*_theo_ vs. MOE).

**FIGURE 4:**
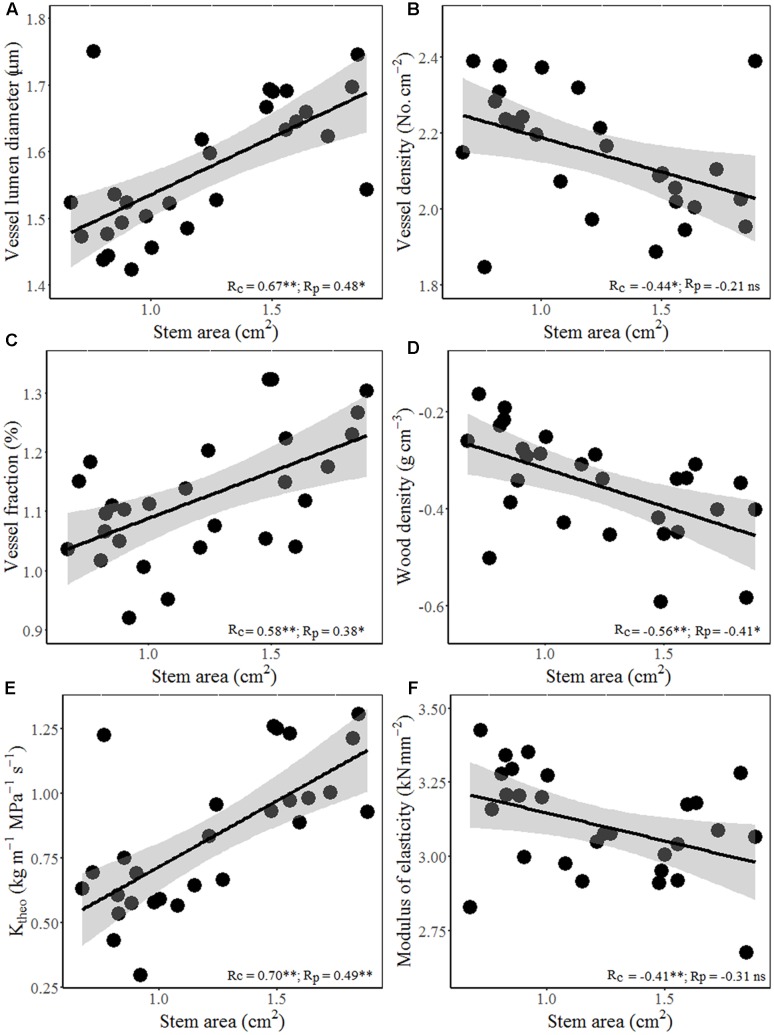
Relationships between stem area and hydraulic architecture and mechanical traits for 28 *Ficus* species. Stem area with **(A)** vessel diameter, **(B)** vessel density (VD), **(C)** vessel fraction (VF), **(D)** WD, **(E)** theoretical hydraulic conductivity (*K*_theo_), and **(F)** MOE. Note the log_10_ scales in panels. Shading areas represent 95% confidence intervals of linear regression. Pearson’s cross-species (*R*_c_) and PIC (*R*_p_) correlation coefficients are shown. *ns*, *p* > 0.05; ^∗^*p* < 0.05; ^∗∗^*p* < 0.01.

**FIGURE 5:**
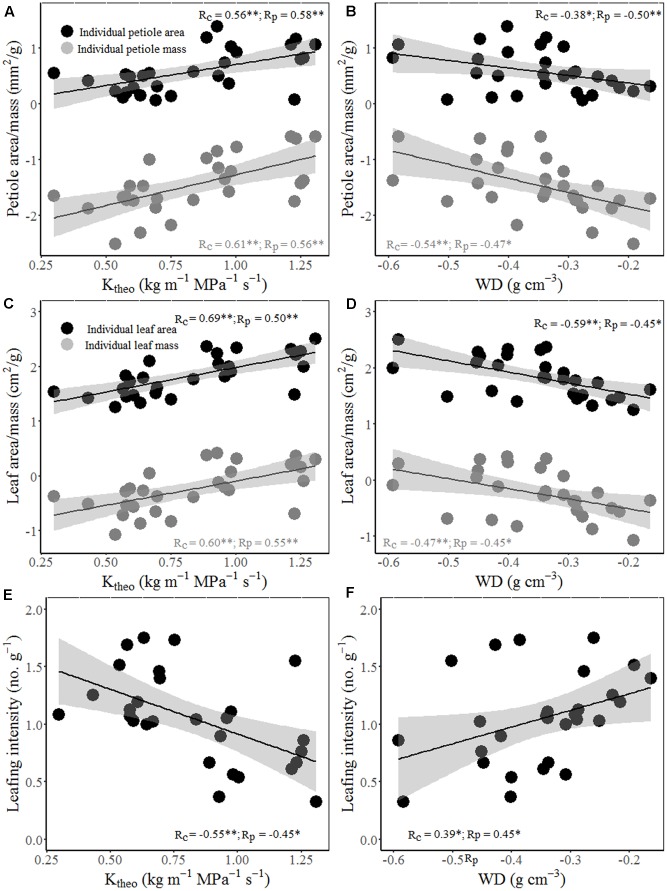
Relationships between theoretical hydraulic conductivity (*K*_theo_) and petiole area/mass **(A)**, LA/mass **(C)** and mass-based LI **(E)**; and between WD and petiole area/mass **(B)**, LA/mass **(D)** and mass-based LI **(F)**. Note the log_10_ scales in panels. Shading areas represent 95% confidence intervals of linear regression. Pearson’s cross-species (*R*_c_) and PIC (*R*_p_) correlation coefficients are shown. ^∗^*p* < 0.05; ^∗∗^*p* < 0.01.

Interestingly, wood hydraulic and mechanical traits varied with stem size of terminal branches (**Figure 4**). For example, stem area correlated positively with vessel lumen diameter (*D*_h_), VF, and theoretical hydraulic conductivity (*K*_theo_), but negatively with VD, WD, and MOE. In other word, thicker stem is more efficient for water transport, but less efficient for mechanical stability. Moreover, significant positive correlations were found between stem theoretical hydraulic conductivity (*K*_theo_) and petiole area/mass and LA/mass for both cross-species means and PICs (**Figure 5**). However, stem WD was negatively correlated with stem, petiole, and leaf dimensions (**Figures 4**, **5**).

## Discussion

We found an isometric relationship between leaf size and stem size, indicating that the increase of total or individual LA/mass is generally proportional to the increase of stem area/mass (**Figures 1A,D** and **Table 2**). These results are consistent with the findings of Brouat et al. (1998) and Li et al. (2008), but in contrast to the others (Preston and Ackerly, 2003; Westoby and Wright, 2003; Sun et al., 2006; Yang et al., 2010). However, the allometrical relationships between lamina size and petiole size (**Figures 1B,E** and **Table 1**) implied that more biomass investment to petiole with a unit increase of lamina size.

**Table 1:** The code and unit of measure of leaf and stem traits of 28 *Ficus* species.

Trait	Abbreviation	Unit
Stem cross-section area	SA	mm^2^
Stem dry mass	SM	g
Vessel lumen diameter	*D*_h_	μm
Vessel density	VD	no. cm^-2^
Vessel fraction	VF	%
Theoretical hydraulic conductivity	*K*_theo_	kg m^-1^ MPa^-1^ s^-1^
Wood density	WD	g cm^-3^
Saturated wood water content	SWC	%
Modulus of elasticity	MOE	kN mm^-2^
Petiole area	PA	mm^2^
Individual petiole mass	IPM	g
Total leaf area	TLA	cm^2^
Individual leaf area	ILA	cm^2^
Total leaf mass	LeM	g
Individual lamina mass	ILM	g
Specific leaf area	SLA	m^2^ kg^-1^
Mass-based leafing intensity	LI	no. g^-1^
Leaf thickness	LT	μm
Upper epidermal thickness	UET	μm
Palisade thickness	PT	μm
Spongy thickness	SP	μm
Lower epidermal thickness	LET	μm
Stomata size	SS	μm
Stomata density	SD	no. mm^-2^


**Table 2:** Standardized major axis regression slopes and 95% confidence intervals (CIs) of the slopes of log–log linear relationships among traits of 28 *Ficus* tree species.

Y	X	*R*^2^	Slope	95% CIs	*P^∗^*
TLA	SM	0.80	0.870	0.728–1.041	0.123
ILA		0.68	0.810	0.647–1.014	0.065
LeM		0.87	0.978	0.845–1.131	0.752
ILM		0.70	0.941	0.756–1.170	0.573
TLA	SA	0.91	1.009	0.892–1.141	0.885
ILA		0.84	0.939	0.800–1.101	0.424
LeM		0.89	1.133	0.991–1.295	0.066
ILM		0.77	1.090	0.900–1.321	0.365
ILA	PA	0.72	0.958	0.775–1.184	0.686
ILM		0.85	1.102	0.942–1.288	0.213
ILA	IPM	0.83	0.720	0.611–0.848	**0.000**
ILM		0.88	0.828	0.721–0.950	**0.009**
TLA	LI	0.69	-0.976	-1.219 to -0.781	0.823
ILA		0.77	-0.908	-1.102 to -0.748	0.318
LeM		0.86	-1.096	-1.277 to -0.941	0.230
ILM		0.87	-1.055	-1.219 to -0.912	0.459
LeM	TLA	0.90	1.123	0.991 to 1.273	0.067
ILM	ILA	0.90	1.150	1.011 to 1.308	**0.034**


*Trait abbreviations and unit of measure are shown in **Table 1**. All the scaling relationships were highly significant (*p* < 0.001). *P^∗^* is the test of isometry (-1 for leaf area (LA)/mass vs. leafing intensity (LI) and 1 for the other pairs of traits). Trait abbreviations are shown in **Table 1**. Bold values indicate significant at *p* < 0.05 level.*

Leaf size scaled negatively and isometrically with LI (number of leaves per unit of stem mass) (**Figures 1C,E**), which demonstrates the generality of the leaf size/number trade-off as found by other studies (Yang et al., 2008; Xiang et al., 2010; Yan et al., 2012). Kleiman and Aarssen (2007) first reported the isometric trade-off for 24 woody species; however, other studies have demonstrated that the scaling relationship between leaf size and LI can be either isometric (Li et al., 2008; Ogawa, 2008; Yang et al., 2008; Milla, 2009) or allometric (Yang et al., 2008; Milla, 2009). Lamina area was positive correlated (allometrically) with lamina mass (slope = 1.15, Supplementary Figure S2), which implied that larger leaves have greater mass per unit LA than smaller leaves, thus a greater cost for building and maintaining a unit of LA (Milla and Reich, 2007; Niklas et al., 2007; Li et al., 2008).

Although more vessels can transport more water, calculated stem theoretical hydraulic conductivity (*K*_theo_) is largely determined by vessel diameter (*D*_h_), because *K*_theo_ increases linearly with VD but with the forth power of *D*_h_, and because of the tight negative relationship between *D*_h_ and VD (**Figure 3**). Significant negative correlation between *K*_theo_ and WD and MOE suggests a trade-off between water transport and mechanical support. Wood composed of closely spaced large vessels will have low density with high hydraulic conductivity, whereas wood composed of small vessels spaced widely within a fiber matrix will be dense with low hydraulic conductivity (Preston et al., 2006; Zanne et al., 2010).

Total LA supported by a twig should be proportional to the xylem conductive area, as predicted by the “pipe model” (Shinozaki et al., 1964). Interestingly, species with bigger size of terminal stems have larger vessels and higher proportion of vessel area in xylem (higher VF), thus higher theoretical hydraulic conductivity (**Figures 4A–C**). These results indicated that higher conductive and lower density woody species grow more rapidly, not only in volume, but also in stem and total leaf mass. Species with higher hydraulic efficiency, expressed by larger *D*_h_, VF, and *K*_theo_, should be able to transport more water and thus deploy a larger total LA per stem (Cavender-Bares et al., 2004). This is also confirmed by a positive correlation between stem hydraulic efficiency and leaf and petiole size (**Figures 5A–D**). Moreover, higher theoretical hydraulic conductivity and lower WD were associated with lower LI (leaf numbers per sapwood mass) (**Figures 5E,F**), which implied that few larger leaves gave a better return for investment on the stem than many smaller leaves (Smith et al., 2017).

In addition to supplying leaves with water and nutrients, twigs also provide mechanical support for static loads and the drag forces exerted on leaves by winds (Niklas and Enquist, 2002). However, species with larger terminal branches tend to have lower WD and mechanical strength (MOE) (**Figures 4D–F**), indicating that bigger branches are less efficient for mechanical strength. The negative relations between leaf size and stem mechanical properties invoke an indirect correlation via plant hydraulics, i.e., species with lower WD have higher hydraulic conductivity per sapwood area as a result of having a higher proportion of stem cross-section taken up by vessel lumen (Martínez-Cabrera et al., 2009), which in turn allow larger LA to be deployed per stem (Wright et al., 2006). For the biomechanical reasons, the relationship between lamina size and petiole mass is allometric rather than isometric (**Figure 1**; Li et al., 2008).

## Conclusion

We provide evidence that leaf–stem size follows an isometric relationship across 28 *Ficus* species growing in a common garden of tropical China. However, large leaves tend to have a larger fractional biomass investment in petioles. Larger stems have wider vessel lumens in the xylem and achieve higher stem specific hydraulic conductivity, but the function of mechanical strength is diminished with less dense wood. The trade-off between hydraulic efficiency and mechanical strength in xylem may shape the leaf–stem allometric relationships. We provide functional interpretation of the relationships between leaves and stem dimensions.

## Author Contributions

Z-XF and P-LF carried out the experiment and collected the data. Z-XF and FS analyzed the data and wrote the first draft of the manuscript. FS, S-BZ, and G-YH contributed to data analysis, writing, and interpretation. All authors contributed critically to the drafts and approved the final version of the manuscript.

## Conflict of Interest Statement

The authors declare that the research was conducted in the absence of any commercial or financial relationships that could be construed as a potential conflict of interest.

## Footnotes

1 http://rsb.info.nih.gov/ij/index.html
