# Supplementary material for: Tradeoff between Stem Hydraulic Efficiency and Mechanical Strength Affects Leaf–Stem Allometry in 28 Ficus Tree Species
Source: Front Plant Sci. 2017 Sep 20;8:1619. doi: 10.3389/fpls.2017.01619 (PMC5611361; doi:10.3389/fpls.2017.01619)

**Supporting Information**

**TABLE S1** List of 28 *Ficus* species, growth form and the Genbank accession numbers. ITS, internal transcribed spacer (ITS); G3pdh, glyceraldehydes-3-phosphate dehydrogenase (G3pdh); H, hemi-epiphytic; NH, non-hemi-epiphytic. Growth and life forms were adapted from Zhu et al. (1996) and Hao (2016).

| Species | Section | Growth form | Life form | Genbank accession  ITS/G3pdh |
| --- | --- | --- | --- | --- |
| *Ficus altissima* | Conosycea | Large tree | H | AY730064/ EU087621 |
| *Ficus annulata* | Conosycea | Large tree | H | JN117618/ EU087622 |
| *Ficus auriculata* | Sycomorus | Small tree | NH | AF165376/ JN117685 |
| *Ficus benjamina* | Conosycea | Tree | H | JN117620/ JN117687 |
| *Ficus callosa* | Oreosycea | Large tree | NH | AY063565/ EF092367 |
| *Ficus binnendijkii* | Conosycea | Tree | H | JN117620/ EF092334 |
| *Ficus concinna* | Urostigma | Large tree | H | JN117626/ JN117692 |
| *Ficus curtipes* | Conosycea | Tree | H | JN117627/ JN117693 |
| *Ficus deltoidea* | Ficus | Tree | H | AY063579/ EF092378 |
| *Ficus drupacea* | Conosycea | Small tree | NH | AY730066/ EF092335 |
| *Ficus elastica* | Conosycea | Tree | H | HM368191/ EF092338 |
| *Ficus fistulosa* | Sycocarpus | Small tree | NH | AY730137/ JN117695 |
| *Ficus glaberrima* | Conosycea | Tree | NH | JN117630/ JN117696 |
| *Ficus hispida* | Sycocarpus | Tree | NH | JN117634/ JN117700 |
| *Ficus langkokensis* | Eriosycea | Small tree | H | JN117638/ JN117703 |
| *Ficus maclellandi* | Conosycea | Tree | H | JN117639/ JN117704 |
| *Ficus microcarp* | Conosycea | Tree | H | JN117640/ JN117705 |
| *Ficus oligodon* | Sycomorus | Tree | NH | JN117641/ JN117706 |
| *Ficus pisocarpa* | Conosycea | Tree | H | JN117643/ JN117707 |
| *Ficus racemosa* | Sycomorus | Large tree | NH | AF165405/ JN126051 |
| *Ficus semicordata* | Sycomorus | Small tree | NH | JN117646/ JN117710 |
| *Ficus stenophylla* | Ficus | Shrub | NH | HQ890713/ HQ890583 |
| *Ficus stricta* | Conosycea | Tree | H | JN117647/ EU087632 |
| *Ficus superba* | Urostigma | Small tree | NH | AF165410/ HQ890554 |
| *Ficus tinctoria* | Sycidium | Tree | H | JN117649/ JN117713 |
| *Ficus variegata* | Sycomorus | Small tree | NH | AF165415/ HQ890563 |
| *Ficus vasculosa* | Oreosycea | Small tree | NH | JN117652/ JN117715 |
| *Ficus virens* | Urostigma | Large tree | H | JN117616/ JN117684 |

**TABLE S2** Hierarchical variance component analysis for the functional traits of the 28 *Ficus* species. Values are ANOVA type I sums of squares, converted to percentages at each level. See Table 1 for trait codes and units of measure.

| Variables | Species | Individual within species | Twigs within individuals | Variables | Species | Individual within species | Twigs within individuals |
| --- | --- | --- | --- | --- | --- | --- | --- |
| SA | 94.6 | 4.0 | 1.4 | ILA | 97.3 | 0.7 | 2.1 |
| SM | 90.6 | 6.5 | 2.9 | ILM | 96.1 | 1.8 | 2.0 |
| D_h_ | 86.7 | 12.2 | 1.1 | SLA | 98.7 | 0.7 | 0.6 |
| VD | 75.6 | 16.8 | 7.6 | LI | 92.9 | 5.5 | 1.7 |
| VF | 78.6 | 16.8 | 4.6 | LT | 99.2 | 0.6 | 0.3 |
| K_theo_ | 84.3 | 14.3 | 1.5 | UET | 99.8 | 0.1 | 0.2 |
| WD | 89.9 | 4.4 | 5.7 | LET | 98.8 | 1.0 | 0.3 |
| SWC | 90.7 | 2.3 | 7.1 | PT | 90.8 | 3.0 | 6.3 |
| MOE | 85.5 | 7.2 | 7.2 | SP | 98.5 | 1.2 | 0.2 |
| PA | 96.6 | 1.9 | 1.5 | SS | 99.3 | 0.5 | 0.1 |
| IPM | 87.8 | 10.9 | 1.3 | SD | 94.3 | 4.7 | 1.0 |

**TABLE S3** Pearson (upper-right) and phylogenetically independent contrast (lower-left) correlations among traits of 28 *Ficus* species. Bold and italic fonts indicate significant correlations at *P* < 0.01 and *P* < 0.05, respectively. See Table 1 for trait codes and units of measure

|  | SA | SM | D_h_ | VD | VF | K_theo_ | WD | SWC | MOE | PA | IPM | ILA | ILM | SLA | LI | LT | UET | LET | PT | SP | SS | SD |
| --- | --- | --- | --- | --- | --- | --- | --- | --- | --- | --- | --- | --- | --- | --- | --- | --- | --- | --- | --- | --- | --- | --- |
| SA |  | **0.91** | **0.67** | *-0.44* | **0.58** | **0.70** | **-0.56** | *0.42* | *-0.41* | **0.86** | **0.86** | **0.92** | **0.87** | -0.21 | **-0.88** | 0.19 | 0.10 | 0.18 | 0.08 | 0.23 | 0.13 | 0.15 |
| SM | **0.90** |  | **0.55** | -0.30 | **0.57** | **0.61** | *-0.42* | 0.27 | -0.34 | **0.85** | **0.81** | **0.83** | **0.84** | -0.34 | **-0.94** | 0.16 | 0.12 | 0.17 | 0.07 | 0.17 | 0.08 | 0.22 |
| Dh | *0.48* | *0.44* |  | **-0.85** | **0.61** | **0.95** | **-0.73** | **0.62** | **-0.50** | **0.49** | **0.59** | **0.66** | **0.54** | 0.12 | **-0.49** | -0.03 | -0.22 | -0.22 | -0.01 | 0.07 | -0.15 | 0.30 |
| VD | -0.21 | -0.11 | **-0.76** |  | -0.10 | **-0.64** | **0.60** | **-0.59** | **0.45** | -0.23 | *-0.40* | *-0.44* | -0.31 | -0.23 | 0.26 | 0.06 | 0.26 | 0.25 | 0.14 | -0.08 | 0.04 | -0.06 |
| VF | *0.38* | *0.47* | 0.33 | 0.35 |  | **0.82** | **-0.53** | 0.35 | -0.32 | **0.54** | **0.48** | **0.58** | **0.54** | -0.07 | **-0.52** | -0.01 | -0.10 | -0.10 | 0.05 | -0.01 | -0.26 | **0.50** |
| K_theo_ | **0.49** | **0.50** | **0.87** | -0.35 | **0.74** |  | **-0.72** | **0.62** | *-0.46* | **0.56** | **0.61** | **0.69** | **0.60** | 0.05 | **-0.55** | -0.02 | -0.19 | -0.19 | -0.00 | 0.04 | -0.21 | *0.41* |
| WD | *-0.41* | *-0.45* | **-0.59** | 0.29 | *-0.46* | **-0.61** |  | **-0.90** | **0.60** | *-0.38* | **-0.54** | **-0.59** | *-0.47* | -0.17 | *0.39* | -0.00 | 0.23 | 0.23 | -0.01 | -0.12 | 0.17 | *-0.40* |
| SWC | 0.35 | 0.33 | **0.49** | -0.35 | 0.27 | *0.45* | **-0.88** |  | **-0.66** | 0.21 | *0.40* | *0.45* | 0.32 | 0.23 | -0.24 | -0.07 | -0.29 | -0.28 | -0.05 | 0.06 | -0.09 | 0.25 |
| MOE | -0.31 | -0.36 | *-0.39* | 0.27 | -0.27 | -0.36 | **0.70** | **-0.75** |  | -0.21 | -0.29 | *-0.40* | -0.24 | -0.30 | 0.24 | -0.03 | 0.15 | 0.12 | 0.10 | -0.12 | 0.03 | -0.10 |
| PA | **0.90** | **0.88** | **0.51** | -0.15 | **0.51** | **0.58** | **-0.50** | 0.37 | -0.22 |  | **0.84** | **0.85** | **0.92** | **-0.55** | **-0.92** | *0.37* | 0.36 | *0.41* | 0.26 | 0.35 | 0.32 | 0.01 |
| IPM | **0.82** | **0.77** | **0.52** | -0.25 | *0.40* | **0.56** | *-0.47* | *0.39* | -0.26 | **0.83** |  | **0.91** | **0.94** | *-0.42* | **-0.87** | 0.25 | 0.15 | 0.16 | 0.28 | 0.28 | 0.12 | 0.26 |
| ILA | **0.86** | **0.76** | **0.50** | -0.26 | 0.37 | **0.50** | *-0.45* | 0.37 | -0.34 | **0.85** | **0.86** |  | **0.95** | -0.21 | **-0.88** | 0.14 | 0.01 | 0.06 | 0.11 | 0.19 | 0.03 | 0.24 |
| ILM | **0.86** | **0.83** | **0.50** | -0.20 | *0.46* | **0.55** | *-0.45* | 0.37 | -0.28 | **0.91** | **0.92** | **0.96** |  | **-0.51** | **-0.93** | 0.30 | 0.21 | 0.25 | 0.25 | 0.31 | 0.20 | 0.15 |
| SLA | *-0.43* | **-0.58** | -0.25 | -0.07 | *-0.44* | *-0.40* | 0.25 | -0.19 | 0.00 | **-0.58** | **-0.59** | -0.34 | **-0.58** |  | **0.51** | **-0.51** | **-0.61** | **-0.60** | *-0.44* | *-0.41* | **-0.55** | 0.22 |
| LI | **-0.89** | **-0.96** | *-0.42* | 0.14 | *-0.40* | *-0.45* | *0.45* | -0.35 | 0.34 | **-0.89** | **-0.83** | **-0.84** | **-0.90** | **0.59** |  | -0.26 | -0.22 | -0.27 | -0.20 | -0.26 | -0.18 | -0.17 |
| LT | *0.48* | *0.43* | 0.22 | -0.13 | 0.09 | 0.17 | *-0.38* | 0.35 | -0.17 | **0.50** | **0.49** | 0.34 | *0.45* | **-0.52** | *-0.47* |  | **0.88** | **0.86** | **0.73** | **0.96** | *0.47* | -0.25 |
| UET | *0.41* | 0.35 | 0.02 | 0.03 | 0.00 | -0.03 | -0.24 | 0.21 | -0.03 | *0.40* | 0.31 | 0.21 | 0.28 | -0.35 | -0.36 | **0.92** |  | **0.92** | **0.63** | **0.76** | **0.61** | *-0.44* |
| LET | **0.56** | *0.47* | 0.10 | 0.00 | 0.11 | 0.08 | -0.26 | 0.23 | -0.12 | **0.51** | *0.39* | 0.33 | *0.41* | *-0.40* | *-0.47* | **0.89** | **0.92** |  | **0.61** | **0.75** | **0.68** | **-0.50** |
| PT | 0.28 | 0.28 | 0.10 | 0.04 | 0.15 | 0.15 | -0.33 | 0.35 | -0.09 | 0.32 | *0.43* | 0.25 | 0.34 | *-0.41* | -0.34 | **0.77** | **0.69** | **0.62** |  | **0.58** | 0.27 | 0.02 |
| SP | *0.48* | *0.42* | 0.29 | -0.24 | 0.05 | 0.20 | *-0.39* | 0.36 | -0.19 | **0.49** | **0.52** | 0.35 | *0.46* | **-0.53** | *-0.46* | **0.97** | **0.85** | **0.83** | **0.63** |  | *0.39* | -0.19 |
| SS | **0.49** | 0.30 | *0.41* | **-0.49** | -0.16 | 0.15 | -0.28 | 0.36 | -0.27 | *0.39* | 0.23 | 0.32 | 0.30 | -0.12 | -0.29 | **0.50** | *0.48* | **0.59** | 0.16 | **0.55** |  | **-.86** |
| SD | -0.02 | 0.21 | -0.08 | *0.45* | **0.56** | 0.27 | -0.11 | -0.05 | 0.08 | 0.13 | 0.31 | 0.05 | 0.19 | *-0.46* | -0.22 | -0.03 | -0.12 | -0.16 | 0.26 | -0.09 | **-0.74** |  |

**FIGURE S1** Phylogenetic relationship of the 28 *Ficus* tree species, based on internal transcribed spaced (ITS) and glyceraldehydes-3-phosphate dehydrogenase (G3pdh) genes. Posterior probability values are listed at the nodes.


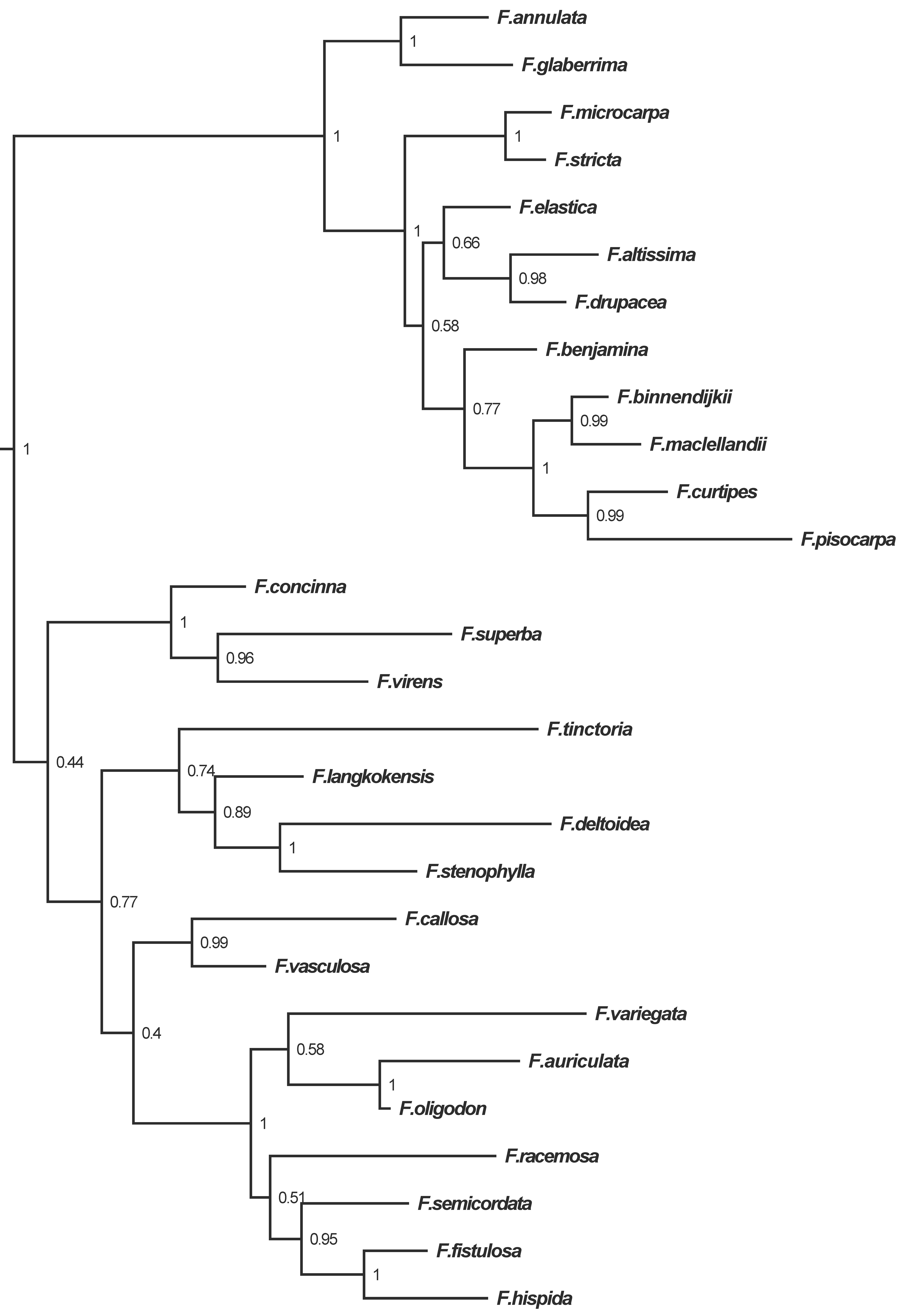


**FIGURE S2** The scaling relationships between lamina mass and lamina area for 28 *Ficus* tree species. The solid lines are the standardized major axis (SMA) regression curves. The regressions do differ significantly from 1.0 for individual lamina area *v.s* mass (dashed lines).


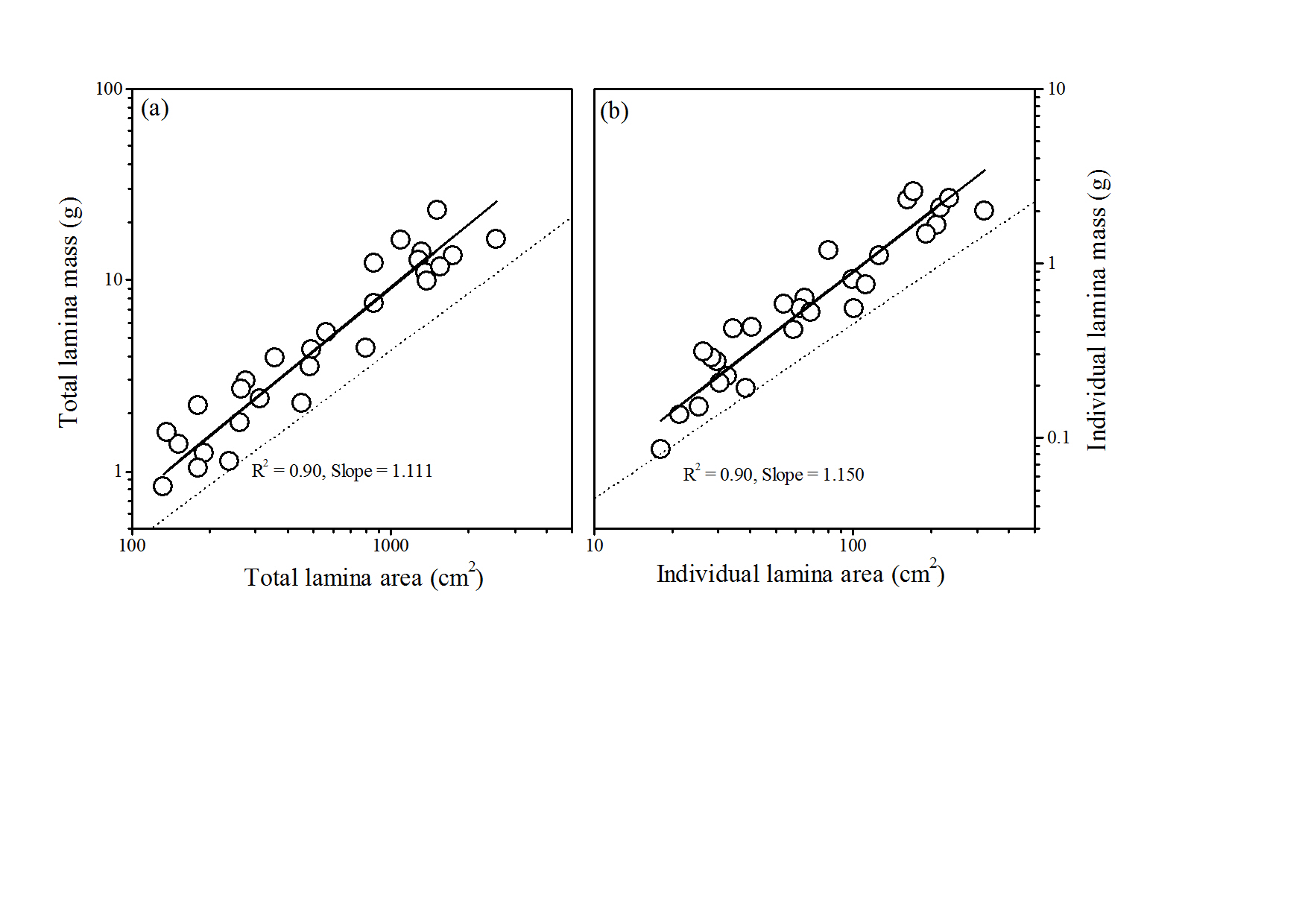


**FIGURE S3** Relationships of specific leaf area (SLA, m^2^ kg^-1^) with tissue thickness (μm) of leaf lamina tissues for 28 *Ficus* tree species. UET: upper epidermal thickness; PT: Palisade thickness; SP: Spongy thickness; LET: lower epidermal thickness; LT: leaf thickness. Shading areas represent 95% confidence intervals of linear regression.


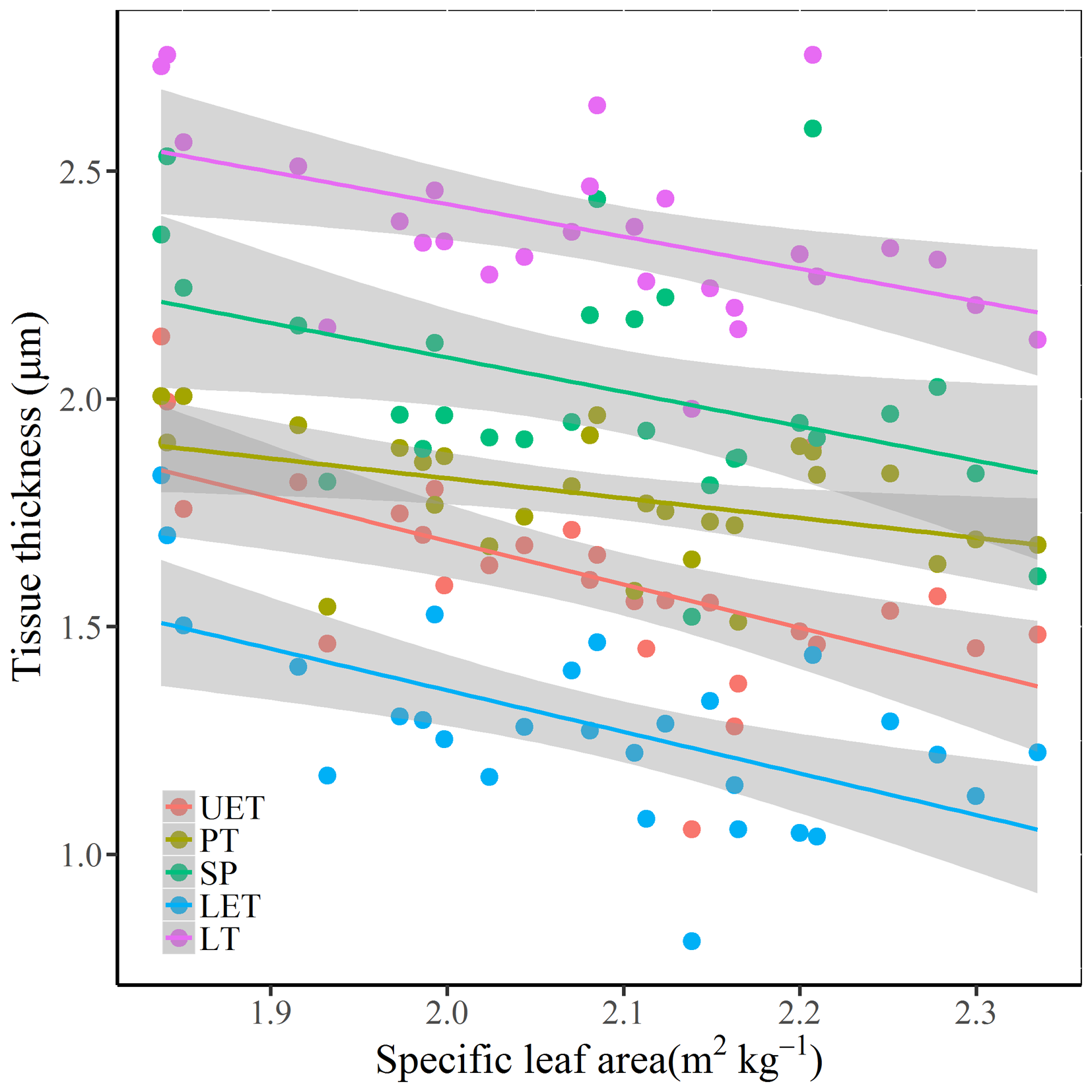


**FIGURE S4** Biplot of trait relationships based on multiple factor analysis (MFA) on phylogenetically independent contrasts of 11 stem/petiole (red) and 13 leaf traits (green) of 28 *Ficus* species. See Table 1 for trait codes and units of measure. Values in parentheses in the axis labels are percentages of variance explained.


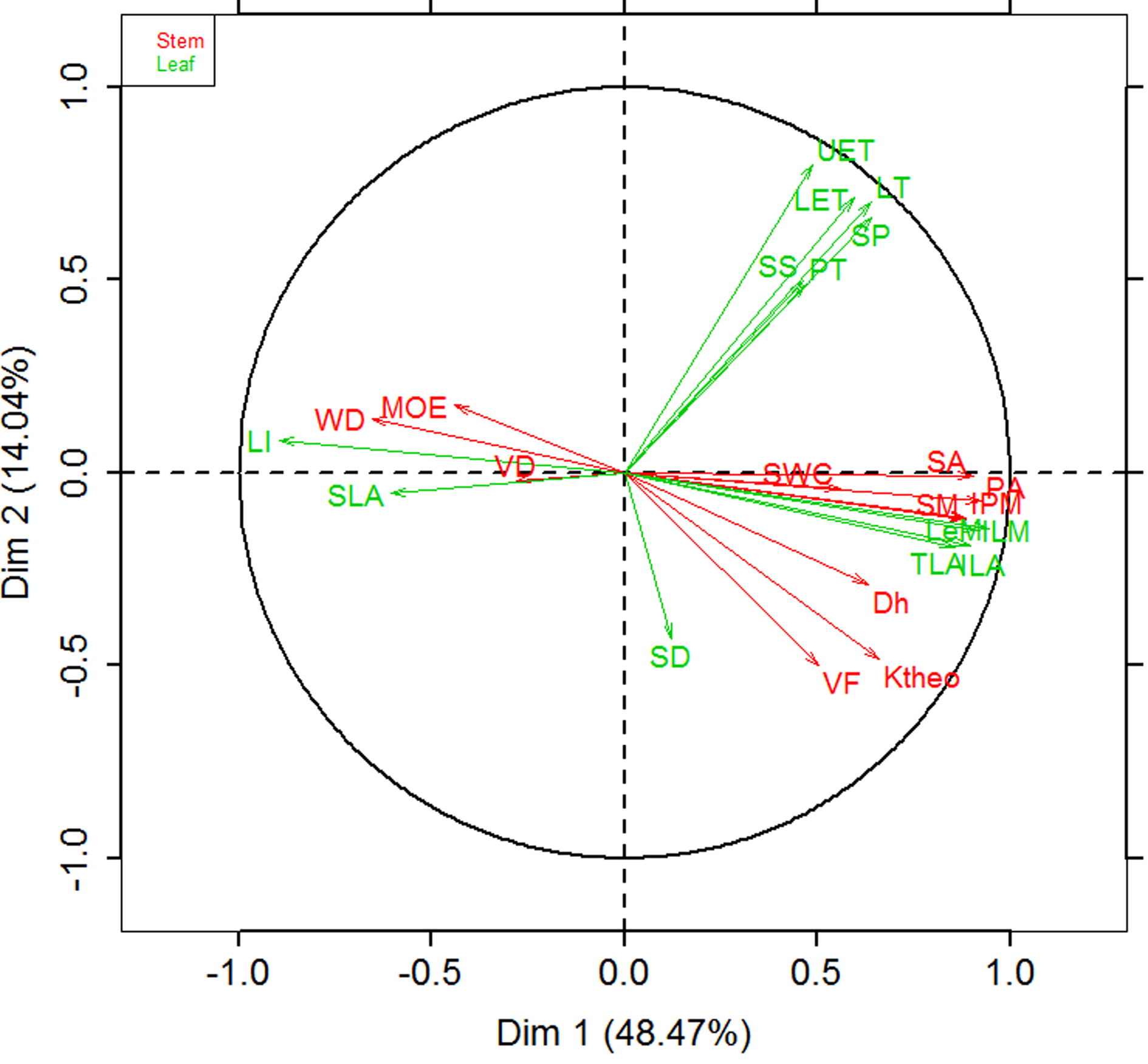

Supplement: Supplementary file 1 [file Data_Sheet_1.doc]
